# IRS-2/Akt/GSK-3*β*/Nrf2 Pathway Contributes to the Protective Effects of Chikusetsu Saponin IVa against Lipotoxicity

**DOI:** 10.1155/2021/8832318

**Published:** 2021-04-03

**Authors:** Lei Wang, Jialin Duan, Na Jia, Meiyou Liu, Shanshan Cao, Yan Weng, Wei Zhang, Jinyi Cao, Ruili Li, Jia Cui, Jingwen Wang

**Affiliations:** Department of Pharmacy, Xijing Hospital, Fourth Military Medical University, Xi'an Shaanxi 710032, China

## Abstract

Chronic hyperlipidemia leads to pancreatic *β*-cell apoptosis and dysfunction through inducing oxidative stress. Chikusetsu saponin IVa (CHS) showed antioxidant and antidiabetic properties in our previous studies; however, its protective effects against lipotoxicity-induced *β*-cell oxidative stress and dysfunction are not clear. This study was designed to investigate the effects of CHS against lipotoxicity-induced *β*-cell injuries and its possible mechanism involved. High-fat (HF) diet and a low dose of streptozotocin- (STZ-) induced type 2 diabetes mellitus (T2DM) model *in vivo* and *β*TC3 cells subjected to 0.5 mM palmitate (PA) to imitate the lipotoxic model *in vitro* were performed. Pancreatic functions, ROS, and antioxidant protein measurements were performed to evaluate the effects of CHS on cell injuries. Protein expression levels were measured by Western blotting. Furthermore, siRNA-targeted Nrf2, PI3K/Akt inhibitor (LY294002), or GSK-3*β* inhibitor (LiCl) was used to investigate the crosstalk relationships between proteins. As the results showed, CHS treatment inhibited apoptosis, promoted insulin release, and reduced oxidative stress. CHS treatment significantly increased the expression of Nrf2 in the cytoplasm and nuclear protein. The antioxidative and benefit effects of CHS were inhibited by siNrf2. The phosphorylation of IRS-2, PI3K, Akt, and GSK-3*β* was markedly increased by CHS which were inhibited by PA. In addition, inhibition of PI3K/Akt or GSK-3*β* with specific inhibitors dramatically abrogated the protective effects of CHS, revealing that the IRS-2/Akt/GSK-3*β* signaling axis was involved in the protective effects of CHS. These results demonstrate that CHS protected *β*TC3 cells against PA-induced oxidative stress and cell dysfunction through Nrf2 by the IRS-2/Akt/GSK-3*β*-mediated pathway.

## 1. Introduction

Diabetes mellitus is the most common chronic disease, which is predicted to affect approximately 592 million adults worldwide by 2030 [1]; among these, type 2 diabetes mellitus (T2DM) accounts for ~90% of diabetes mellitus cases. T2DM is characterized by progressive impairment of insulin secretion and insulin resistance. In nondiabetic subjects, insulin secretion is precisely controlled by glucose and other stresses such as amino acids, catecholamines, and intestinal hormones. However, these regulation effects are destroyed by chronic and long-term hyperglycemia and hyperlipidemia, which will induce pancreatic *β*-cell dysfunction and death. High-fat diet (HFD), obesity, and sedentary lifestyle are important risk factors to induce pancreatic *β*-cell injuries [2, 3]. Prolonged exposure of pancreatic *β*-cells to elevated levels of free fatty acids (FFAs) decreases *β*-cell sensitivity to stimulation and increases *β*-cell dysfunction and *β*-cell mass lose. This is defined as lipotoxicity and contributes to the pathogenesis of T2DM [4–7].

Oxidative stress is a major contributor to FFA-induced pancreatic *β*-cell damage and dysfunction, and it plays an essential role in the onset and progression of diabetes and its complications [8–10]. Oxidative stress has been shown to damage the structural and functional integrity of the *β*-cell by either directly modifying or initiating chain reactions that cause extensive oxidative damage to DNA, proteins, and lipids [11–13]. Pancreatic *β*-cells are susceptible to oxidative stress due to the intrinsically low levels of antioxidant enzyme expression and the overproduction of reactive oxygen species (ROS) within the cell [14]. Thus, attenuating oxidative stress and raising antioxidant enzyme activities play a vital role in protection against lipotoxic *β*-cell failure.

There are many factors associated with the cellular response to oxidative stress; among these, the transcriptional factor nuclear factor-erythroid 2-related factor-2 (Nrf2) is a major regulator of cellular and organismal defense mechanism that binds to antioxidant response element- (ARE-) dependent genes against endogenous and exogenous oxidative stresses [15, 16]. Hundreds of Nrf2/ARE-driven genes are known to be exploited for detoxification and antioxidant defense [17]. Thus, targeting Nrf2 has emerged as an attractive therapeutic strategy for *β*-cell protection. Nrf2 can be activated by upstream kinases, such as phosphoinositide 3-kinase/protein kinase B (PI3K/Akt) and glycogen synthase kinase-3*β* (GSK-3*β*), which are downstream of insulin receptor substrate-2 (IRS-2), a key marker in the insulin-related metabolic process [18–21]. Several studies demonstrated that GSK-3*β* acts as an important negative regulator and plays a central role in the PI3K/Akt pathway [22]. Knockout of GSK-3*β* in *β*-cells leads to expansion of *β*-cell mass accompanied by increased proliferation and decreased apoptosis through the insulin PI3K/Akt signaling pathway [23]. These findings indicated the essential role of the IRS-2/Akt/GSK-3*β*/Nrf2 pathway in *β*-cell protection.

Saponin has a high potential for antidiabetic remedy and hypotriglyceridemic and hypocholesterolemic effects. Chikusetsu saponin IVa (CHS) is the most-potent antioxidant among the triterpenoid saponins isolated from the Chinese herb *Aralia taibaiensis*. Our studies showed that CHS pretreatment promoted the inhibitory phosphorylation of GSK-3*β* and protected the brain from oxidative stress [24]. In pancreatic *β*-cells, we have proven that CHS could increase insulin secretion [25], but whether CHS had protective effects against pancreatic *β*-cell injuries and its possible mechanism is still unknown. Thus, the present study is aimed at determining the effect of CHS on FFA-induced pancreatic *β*-cell dysfunction and exploring its mechanisms regarding oxidative stress.

## 2. Materials and Methods

### 2.1. Drugs and Reagents

Chikusetsu saponin IVa (CHS, purity > 98%) was separated from *Aralia taibaiensis* and acquired from the New Drug Research and Development Center, Fourth Military Medical University.

Dulbecco's modified Eagle's medium (DMEM), trypsin, penicillin-streptomycin (penstrep), and fetal bovine serum (FBS) were purchased from GIBCO (Life Technologies, Grand Island, NY). Cell counting kit 8 (CCK-8) assay was purchased from 7sea Biotech (Shanghai, China), Fluorescein Annexin V-FITC/PI double labeling kit was purchased from the Nanjing Jiancheng Bioengineering Institute (Nanjing, China), insulin radioimmunoassay (RIA) kit was purchased from the Institute of Atomic Energy (Beijing, China), and streptozotocin (STZ), LiCl, LY294002, 2,7-dichlorofluorescein diacetate (DCFH-DA), and dihydroethidium (DHE) were purchased from Sigma-Aldrich Co. (St. Louis, MO, USA).

Total cholesterol (TC), triglyceride (TG), low density lipoprotein cholesterol (LDL-C), high density lipoprotein cholesterol (HDL-C), serum levels of free fatty acid (FFA), superoxide dismutase (SOD), malondialdehyde (MDA), and glutathione peroxidase (GSH-Px) assay kits were purchased from Jiancheng Bioengineering Institute (Nanjing, China). Palmitate was obtained from Shanghai Macklin Biotechnology (Shanghai, China). Fatty acid-free bovine serum albumin (BSA) was obtained from Shanghai Yeasen Biotechnology (Shanghai, China).

Rabbit anti-Nrf2 (#12721, 1 : 1000, Cell Signaling Technology), rabbit anti-IRS-2 (#3089, 1 : 1000, Cell Signaling Technology), rabbit anti-Akt (#9272, 1 : 1000, Cell Signaling Technology), rabbit anti-P-Akt (#4060, 1 : 2000, Cell Signaling Technology), rabbit anti-GSK-3*β* (#12456, 1 : 1000, Cell Signaling Technology), rabbit anti-P-GSK-3*β* (Ser9) (#5558, 1 : 1000, Cell Signaling Technology), rabbit anti-PDX-1 (#5679, 1 : 1000, Cell Signaling Technology), rabbit anti-SOD1 (#2770, 1 : 1000, Cell Signaling Technology), rabbit anti-*β*-actin (#4970, 1 : 1000, Cell Signaling Technology), and rabbit anti-Lamin B (#12255, 1 : 1000, Cell Signaling Technology).

## 3. *In Vivo*

### 3.1. Animals and Treatments

Male Sprague Dawley rats (160-180 g) were obtained from the Experimental Animal Center of the Fourth Military Medical University. All of the protocols in this study were approved by the Ethics Committee for Animal Experimentation (No. 20170510) and performed according to the Guidelines for Animal Experimentation of the Fourth Military Medical University and the National Institute of Health Guide for the Care and Use of Laboratory Animals (NIH Publication No. 80-23) revised in 2011. The animals were housed at 22-25°C temperature under a 12-hour light/dark cycle and given free access to food and water. After one week of acclimatization, animals were randomly assigned into a control group and a diabetes group. The animals had free access to water and rodent chow—either standard rat chow consisting of 10% fat, 20% protein, and 70% carbohydrate or a high-fat diet (HFD) consisting of 45% fat, 20% protein, and 35% carbohydrate (4.73 kcal/gm, cat no. HF45, Dyets Biotechnology Co., Ltd., Wuxi, China)—for 4 weeks then fasted overnight and given STZ at a dose of 35 mg/kg which was freshly dissolved in 100 mM citrate buffer (pH 4.4) through intraperitoneal injection [26]. One week after the STZ injection, fasting blood glucose levels were measured from a vein using an Accu-Chek Performa glucometer (Roche Diagnostics, Shanghai, China); rats with plasma glucose concentration more than 16.7 mM were selected for the subsequent experiments. All the rats were continually fed with the same diet until the end of the study.

The rats were randomly allocated into five groups: normal control group (CON), model of diabetes control group (MOD), and diabetic group treated with 45, 90, and 180 mg/kg CHS. CHS was dissolved in sterile saline and administered once per day by gastric gavage for 4 consecutive weeks; the equivalent volume of sterile saline solution was administered to the control. After experiments, rats were sacrificed by a lethal dose of sodium pentobarbital (100 mg/kg; Sigma), and the blood and tissue samples were collected and stored at -80°C for further analysis.

### 3.2. Oral Glucose Tolerance Test (OGTT)

At the end of the trial, OGTT was used to measure the glucose-induced insulin secretion and its mediated glycemic alterations. After fasting overnight, rats were orally administered with glucose (2 g/kg), then the blood glucose level was determined at 0, 30, 60, and 120 min after glucose administration. The blood glucose level collected from the tail vein was determined by using an Accu-Chek glucometer.

### 3.3. Biochemical Analyses

After OGTT, rats were anesthetized by sodium pentobarbital and blood was collected from the aorta ventralis. Serum samples were obtained by centrifuging the blood at 1500 g for 15 minutes. TC, TG, LDL-C, HDL-C, and FFA were measured using commercially available kits (Nanjing Jiancheng Bioengineering Institute, Nanjing, China) according to the manufacturer's instructions. The serum insulin level in every group was determined by the insulin ELISA kit (Beyotime, Shanghai, China).

### 3.4. Hematoxylin and Eosin (HE) Staining

The pancreases were dissected and fixed in 10% buffered formalin (pH 7.4), dehydrated in graded (50-100%) alcohol, then processed and embedded in paraffin blocks. The blocks were cut into 5 *μ*m paraffin sections and stained routinely with HE stain for photomicroscopic assessment.

### 3.5. ROS Measurement

The ROS generation was measured by DCFH-DA; DCFH-DA passively diffuses into cells and is deacetylated by cellular esterases to yield nonfluorescent 2′,7′-dichlorofluorescein (DCFH) which then reacts with ROS to form the fluorescent product 2′,7′-dichlorofluorescin (DCF). Briefly, the whole pancreatic tissues from different groups were homogenized on ice with the lyse buffer (*w*/*v* = 1 : 10), and 25 *μ*M DCFH-DA was added to the homogenates. After incubation for 30 min, changes in fluorescence values were measured at an excitation wavelength of 486 nm and emission wavelength of 530 nm. Accumulation of DCF was measured using a spectrofluorometer (Shimadzu Corp., Japan). The ROS level was expressed as the fold of the fluorescence intensity in the CON group.

### 3.6. Determination of SOD, GSH-Px, and MDA

After the animal was sacrificed, the pancreas was dissected out and homogenized with the lyse buffer (*w*/*v* = 1 : 10). Then, the homogenate was centrifuged at 12000 g for 15 min (at 4°C), and the supernatant was isolated and preserved at -80°C for the determination of MDA, SOD, and GSH-Px. The pancreatic content of the lipid peroxidation products, MDA, was measured by color changes in thiobarbituric acid reactive substances (TBARS) at 532 nm, and the concentration of TBARS was calculated by referring to the standard curve. SOD activity was assayed at 450 nm on the basis of its ability to inhibit the production of formazan dye resulting from the reaction of WST-1 and superoxide anion. The activity of GSH-Px was measured at 412 nm on the basis of the rate of oxidation of reduced glutathione to oxidized glutathione by H_2_O_2_ under the catalysis of GSH-Px.

## 4. *In Vitro*

### 4.1. Cell Culture and Treatments

The *β*TC3 cells were obtained from the China Center for Type Culture Collection (Shanghai, China) and cultured in RPMI 1640 medium supplemented with 10% fetal bovine serum, 100 IU/ml penicillin, and 100 IU/ml streptomycin under 95% O_2_/5% CO_2_. The medium was changed every two days. Palmitate (PA) was dissolved in ethanol to make stock solution at 100 mM, and 5 mM PA was mixed with 5% fatty acid-free BSA in serum-free RPMI 1640 medium to make a final concentration of 0.5 mM palmitate/0.5% BSA. The control group containing the same concentration of BSA was prepared in the same way. Cells were divided into five groups: normal control group (CON), cells treated with 0.5 mM PA for 24 h (MOD), and cells pretreated with CHS (10, 20, 40 *μ*M) or glibenclamide (Gli) for 24 h and treated with PA for another 24 h. For some experiments, the LY294002 (20 *μ*M) or LiCl (20 mM) were pretreated with CHS for 24 h.

### 4.2. Cell Viability Assay

Cell viability was measured using CCK-8 according to the manufacturer's protocol. The *β*TC3 cells were seeded on 96-well plates at a density of 1 × 10^4^ cells per well. The cells were pretreated with various concentrations of CHS (10, 20, and 40 *μ*M) or Gli for 24 h, followed by incubation with vehicle or 0.5 mM PA for 24 h. After cultivation, 10 *μ*l CCK-8 solution was added to each well, and the plates were incubated at 37°C for 2 h; the absorbance at 450 nm was determined using a microplate reader.

### 4.3. Detection of Apoptosis

The cell apoptosis was measured with the Annexin V-FITC/propidium iodide (PI) apoptosis detection kit. The *β*TC3 cells were seeded at 2 × 10^5^ cells per well into 6-well plates and cultured at 37°C for 24 h. After different treatments, cells were resuspended in the binding buffer, and FITC-conjugated Annexin V and PI were added and the mixtures were incubated in the dark for 10 min. Annexin V-FITC and propidium iodide fluorescence were assessed using a flow cytometer (BD, USA).

### 4.4. Glucose Stimulated Insulin Secretion (GSIS)

The *β*TC3 cells were plated onto 24-well culture plates with a cell density at 2 × 10^5^/well; after different treatments, the cells were washed gently by phosphate buffer for three times and then preincubated in KRBH buffer supplemented with 0.1% BSA at 37°C for 1 h. Cells were subsequently incubated in the same buffer containing 3 or 27.8 mM glucose for another 30 min at 37°C. The culture medium in each well was collected for insulin release detection by an insulin RIA kit.

### 4.5. Measurement of Cellular ROS Production

Intracellular ROS levels in *β*TC3 cells were assayed using DCFH-DA and DHE fluorescent probes. The *β*TC3 cells (5 × 10^3^/well) were cultured on glass coverslips for 24 h and subjected to different treatments. Then, cells were washed twice with PBS and incubated with DCFH-DA (20 *μ*M) or DHE (10 *μ*M) for 30 min at 37°C in darkness. The DCF and DHE fluorescence intensity stained intracellular ROS were imaged using a laser confocal microscope (Nikon C2).

### 4.6. Determination of SOD, GSH-Px, and MDA *In Vitro*

For the measurement of SOD, GSH-Px, and MDA, cells were plated into 6-well plates at approximately 5 × 10^5^/well. After different treatments, cells were collected and lysed by the lyse buffer, then centrifuged at 12000 g for 10 min, 4°C. The supernatants were collected for the detection of SOD, GSH-Px, and MDA. For MDA measurement, samples were reacted with the TBA and boiled for 15 min; after being cooled to room temperature, the absorbance was determined at 532 nm. For the detection of SOD and GSH-Px, samples were mixed with reagents as per the manufacturer's protocol, and the absorbance was determined at 560 nm for SOD and 412 nm for GSH-Px.

### 4.7. siRNA Transfection

Nrf2-specific short interfering RNA (siRNA) molecules were synthesized by the Shanghai Genechem Company. The transfection was performed using Lipofectamine 2000, and cells were transfected for 48 h at 37°C. The transfection efficiency was confirmed by Western blotting. After transfection, cells were treated with CHS with or without PA according to the protocols.

### 4.8. Nuclear and Cytosolic Fractionation

Nuclear and cytoplasmic proteins were extracted using the Nuclear Extraction Kit according to the manufacturer's protocol (Beyotime, Shanghai, China). Briefly, cells were homogenized in Cytoplasmic Extraction Reagent A (CER-A) buffer by a homogenizer and incubated on ice for 15 min, then added to a Cytoplasmic Extraction Reagent B (CER-B) buffer and incubated on ice for 1 min. Samples were centrifuged at 15000 rpm for 5 min at 4°C, the supernatant solution was collected (cytoplasmic fraction), and pellets containing nuclei were suspended in a nuclear extraction reagent. After thorough vortexing for 30 min with 1 min break for every 30 s, samples were centrifuged at 15000 rpm for 10 min at 4°C, and the supernatant solution was collected (nuclear fraction).

### 4.9. Western Blotting

Nuclear and cytoplasmic protein extractions were performed after difference treatments in *β*TC3 cells and pancreatic tissues and the protein concentrations determined by the BCA protein assay reagent kit. Equal amounts of protein (30 *μ*g) were separated by sodium dodecyl sulfate-polyacrylamide gel electrophoresis (SDS-PAGE) and transferred onto polyvinylidene difluoride (PVDF) membranes. Membranes were blocked with 5% (*v*/*v*) nonfat dry milk in TBS-T for 2 h at room temperature and then incubated overnight at 4°C with the following primary antibodies: anti-Nrf2, IRS-2, P-IRS-2, Akt, P-Akt, GSK-3*β*, P-GSK-3*β*, SOD1, and *β*-actin. Then, membranes were washed and incubated with secondary antibodies (goat anti-rabbit, 1 : 10000, Cell Signaling Technology) for 1 h at room temperature and visualized using an enhanced chemiluminescent substrate (Thermo Fisher Scientific). The densities of membranes were scanned and quantified with image analysis systems (Bio-Rad, USA).

### 4.10. Nrf2 Transcription Activity

The nuclear protein extraction method was the same with Western blot. The activation of Nrf2 was measured by an ELISA kit (ab207223, Abcam), and the absorbance value was detected at OD 450 nm.

### 4.11. Statistical Analysis

Data from individual experiments were expressed as mean ± SEM. One-way ANOVA followed by Tukey's test was performed using GraphPad Prism version 5.0 (GraphPad Software, La Jolla, CA, USA). *P* < 0.05 was considered to be statistically significant.

## 5. Results

### 5.1. Effects of CHS on Glucose Tolerance and Lipid Parameters in Diabetic Rats

**Figure 1:**
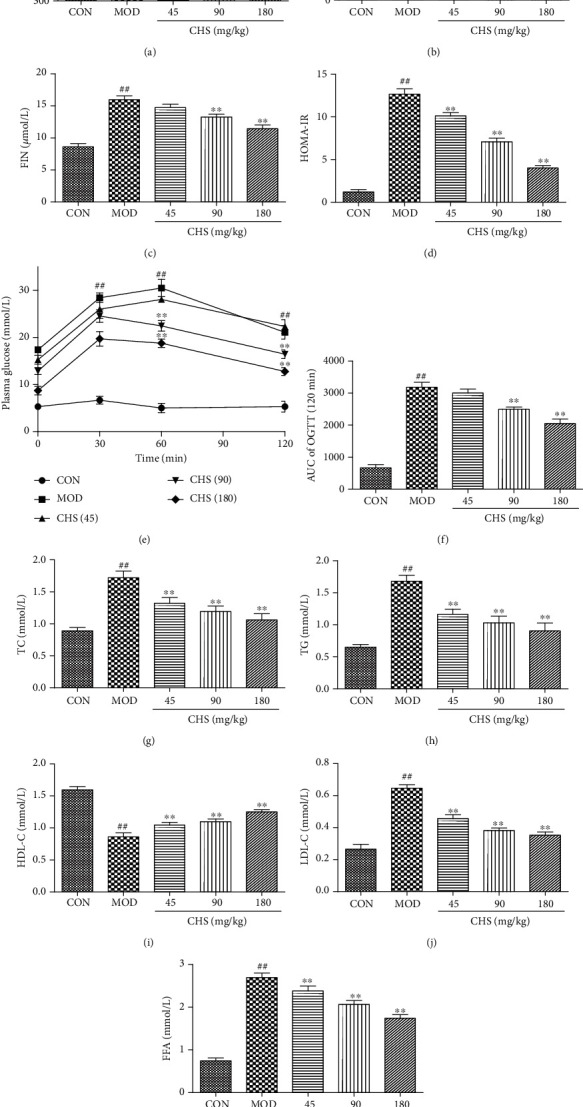
Effect of CHS on plasma glucose and lipid levels. T2DM model was induced by HF and STZ; 45, 90, and 180 mg/kg CHS were given through intragastric administration one time every day for 4 consecutive weeks. (a) Body weight, (b) fasting blood glucose (FBG) levels, (c) fasting insulin (FIN) levels, (d) HOMA-IR, (e) glucose tolerance test (OGTT), (f) AUC of OGTT, (g) plasma TC, (h) TG, (i) HDL-c, (j) LDL-c, and (k) FFA were measured accordingly. The columns and errors bars are presented as means ± SEM. ^##^*P* < 0.01 and ^#^*P* < 0.05 compared with the CON group. ^∗∗^*P* < 0.01 and ^∗^*P* < 0.05 compared with the MOD group.

Physiological parameters were collected to evaluate the changes of the rats in the control and experimental groups. The results show in Figure 1(a) a gradual increase in body weight in the control rats, while the body weight was significantly decreased in the MOD group when compared with the control rats (*P* < 0.05). Treatments with CHS at the dose of 90 mg/kg and 180 mg/kg significantly increased the body weight which was compared with that of MOD group (*P* < 0.05). In MOD rats, the FBG levels (Figure 1(b)) and FIN levels (Figure 1(c)) were increased compared with those of control rats (*P* < 0.05) and decreased by CHS administration significantly (*P* < 0.05). The calculated HOMA-IR index showed a significant increase in the DM group which compared with the control group (*P* < 0.05). The HOMA-IR index was significantly decreased after CHS treatments (Figure 1(d)).

To evaluate the metabolic abilities in diabetic rats, OGTT was performed. As the results show in Figure 1(e), the basal plasma glucose level in the HFD-treated group (MOD) was significantly higher than that in the CON group; after 2 h of glucose administration, the doses of 45, 90, and 180 mg/kg CHS showed significant lower plasma glucose level compared to those of the MOD group (Figure 1(e)). The AUC calculated from Figure 1(e) also shows that T2DM induced glucose homeostasis deterioration which is evident from the large increase of AUC (Figure 1(f)). CHS at the dose of 90 mg/kg and 180 mg/kg significantly decreased the AUC value which is compared with that of the MOD group (*P* < 0.05). It was revealed that CHS-treated groups improved the tolerance for glucose suggesting increased peripheral utilization of glucose.

Then, effects of CHS on the lipid levels were measured. As shown in Figures 1(b)–1(f), the rats in the MOD group showed higher levels of TC, TG, LDL-C, and FFA and lower levels of HDL-C compared with those in the CON group, while treatment with CHS significantly restored all the changes in the lipid profile and FFAs compared to the MOD group. These results indicated that CHS could improve the glucose and lipid metabolism in diabetic rats.

### 5.2. Effects of CHS on Histological Changes of the Pancreas

**Figure 2:**
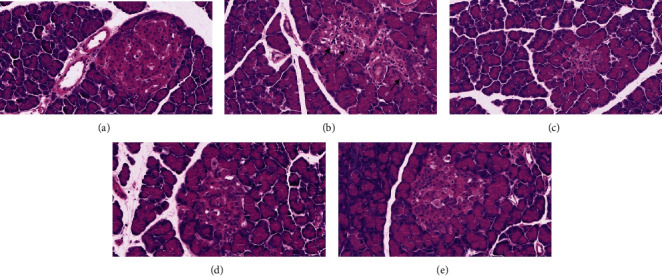
Photomicrographs of sections of the pancreas stained by H&E. By the end of the 4-week treatment, pancreases were harvested from different experimental groups, and the sections were stained with HE: (a) normal rats treated with vehicle alone; (b) diabetic rats treated with vehicle alone; (c) diabetic rats treated with CHS (45 mg/kg); (d) diabetic rats treated with CHS (90 mg/kg); (e) diabetic rats treated with CHS (180 mg/kg). Images (400x) are representative of pancreas sections from each group.

Histopathologic examinations of the pancreas in rats of experimental groups are shown in Figure 2; the CON group showed the normal appearance of islet cells in the pancreas and no histological change was found, while in the MOD group, an apparent reduction was observed in the size and number of islets and ambiguity of their verges. Karyolysis of the nuclei, vacuolation, and invasion of connective tissues were found in the model group, whereas after CHS treatment, there was obvious amelioration in histological signs. CHS-treated groups showed increased volume and number of islets, dark nucleated cells could also be seen, and rats that received CHS at a dose of 180 mg/kg showed almost normal cell morphology.

### 5.3. Effects of CHS on the Levels of Oxidative Stress *In Vivo*

**Figure 3:**
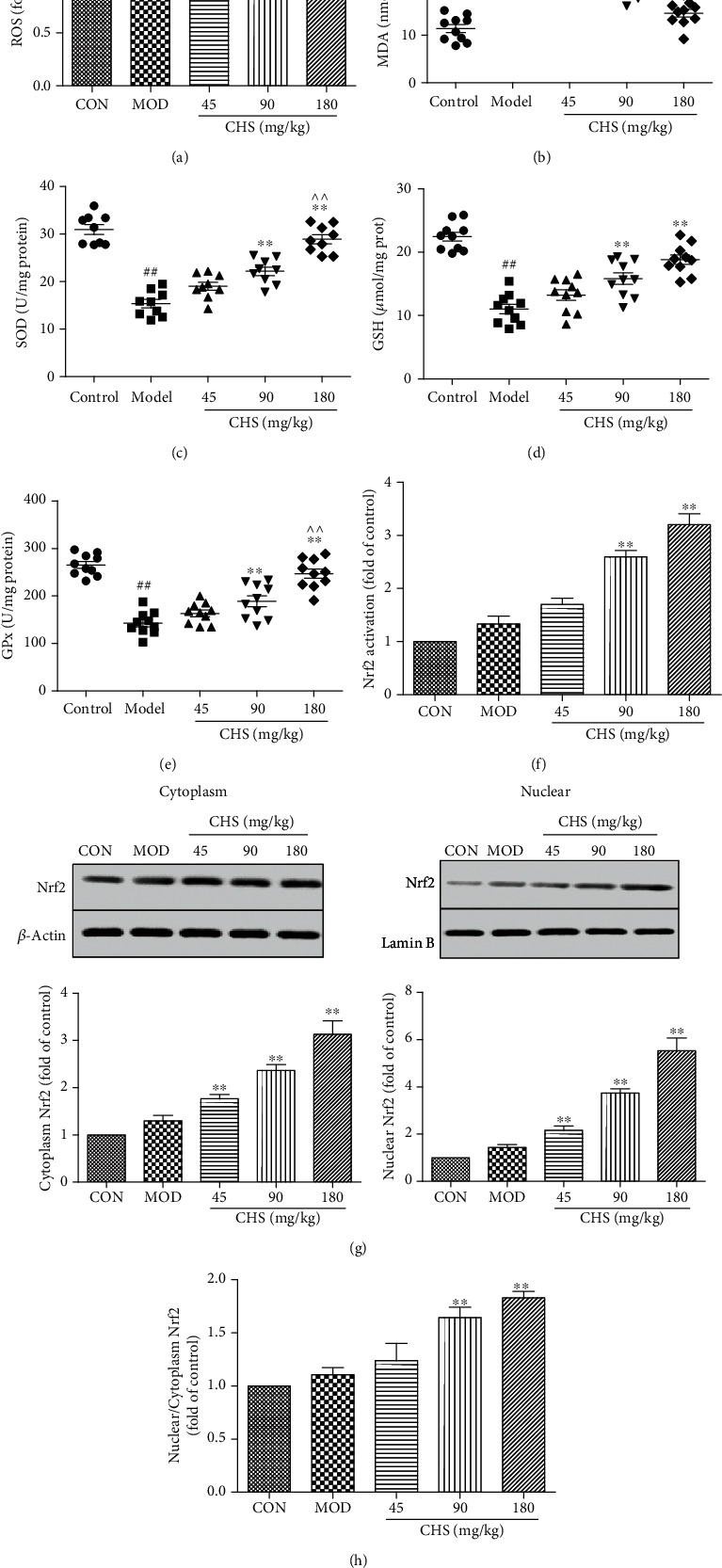
Effect of CHS on the activities of oxidative stress-related markers in rat pancreas after 4 weeks of intervention: (a) ROS level; (b) MDA level; (c) SOD activity; (d) GSH activity; (e) GPx activity; (f) the Nrf2 transcriptional activity; (g) effects of CHS on the expression levels of Nrf2 in cytoplasm and nuclear protein; (h) the ratio of nuclear/cytoplasm Nrf2 levels. The columns and error bars are presented as means ± SEM (*n* = 10). ^##^*P* < 0.01 compared with the CON group. ^∗∗^*P* < 0.01 and ^∗^*P* < 0.05 compared with the MOD group.

ROS in the pancreas was measured by a ROS measurement kit. As the results show in Figure 3(a), ROS was increased in the model group, and CHS inhibited the ROS levels in the 90 and 180 mg/kg treatment groups. MDA, an important indicator for judging lipid peroxidation, was significantly increased in diabetic rats (MOD group); after treatment with CHS, the level of MDA (Figure 3(b)) was significantly decreased compared with the MOD group (*P* < 0.05). The activities of antioxidant enzymes (SOD, GSH, and GPx) in the pancreas of the MOD group were found to be significantly reduced when compared with the CON group, while in CHS-treated groups, SOD, GSH, and GPx were found to be increased (Figures 3(c)–3(e)). The expression levels of Nrf2 in the cytoplasm and nuclear protein were also measured. As the results show in Figure 3(g), CHS treatment significantly increased the expression and translocation of Nrf2 in the islet tissue (Figure 3(g)). In addition, CHS increased the ratio of nuclear/cytoplasm levels (Figure 3(h)) and Nrf2 transcriptional activity in the nuclear protein (Figure 3(f)).

### 5.4. Effects of CHS on PA-Induced Cytotoxicity

**Figure 4:**
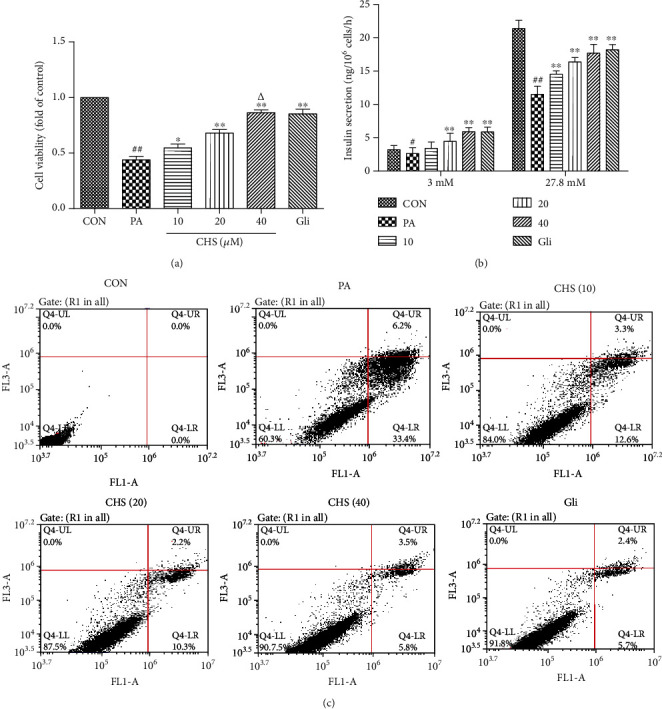
Protective effects of CHS on palmitate-induced lipotoxicity in *β*TC3 cells. *β*TC3 cells were incubated with CHS or Gli for 24 h, then the cell viability (a) was measured by CCK-8, the glucose stimulated insulin secretion (b) was measured by an insulin RIA kit, and the cell apoptosis rates (c) were measured by an Annexin V-FITC/PI double-labeled kit using flow cytometry. The columns and errors bars are presented as means ± SEM (*n* ≥ 5). ^##^*P* < 0.01 and ^#^*P* < 0.05 compared with the CON group. ^∗∗^*P* < 0.01 and ^∗^*P* < 0.05 compared with the PA group. ^△△^*P* < 0.01 and ^△^*P* < 0.05 compared with the CHS (20 *μ*M) group.

To determine whether CHS could protect *β*-cells from PA-induced injury, a CCK-8 assay was performed to measure the cell viability *in vitro*. Various concentrations of CHS were initially tested for cytotoxic activity in *β*TC3 cells. Preliminary experiments demonstrated that CHS was noncytotoxic till 100 *μ*M; pharmacological doses of CHS (10-40 *μ*M) were chosen for further study. As compared with the CON group, exposure to PA decreased *β*TC3 cell viability, which was significantly attenuated by CHS treatment (Figure 4(a)).

To determine whether CHS protection of *β*-cell viability is associated with the improved cellular function, we measured GSIS in *β*TC3 cells exposed to 0.5 mM PA for 24 h. As shown in Figure 4(b), PA incubation significantly attenuated GSIS in response to 27.8 mM glucose. Treatment with CHS prevented PA-induced impairment of GSIS.

Cell apoptosis was also determined by flow cytometric analysis. As shown in Figure 4(c), PA significantly increased the apoptosis rate in *β*TC3 cells compared with the CON group. Compared with the PA group, CHS decreased apoptosis in rats, showing the inhibition effects on PA-induced cell apoptosis.

### 5.5. Effects of CHS on the Level of Oxidative Stress *In Vitro*

**Figure 5:**
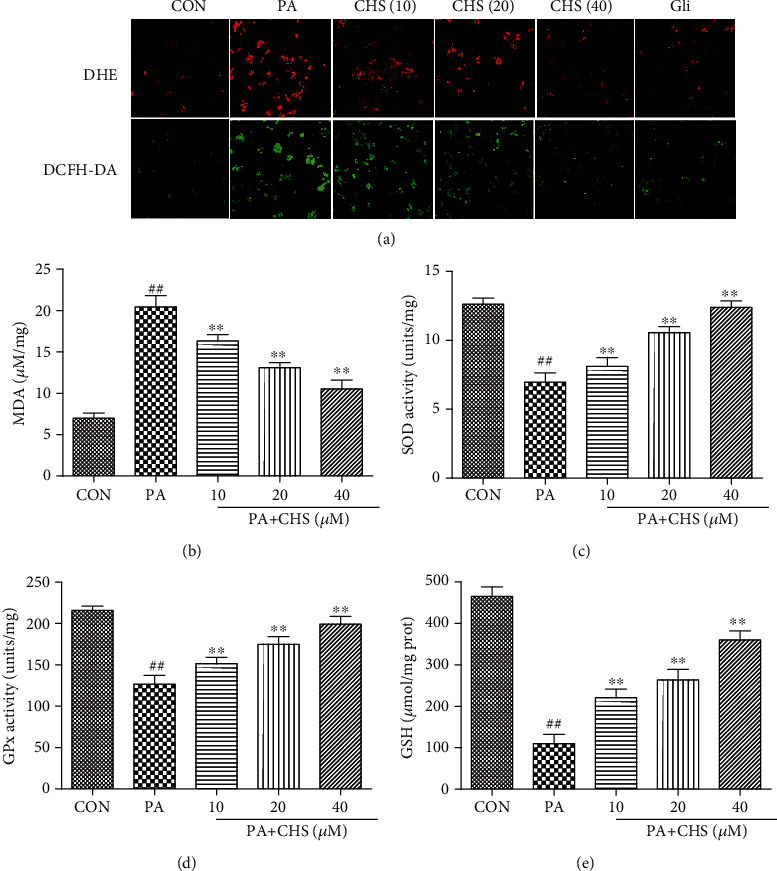
The antioxidative effects of CHS *in vitro*. (a) The effects of CHS on palmitate-induced ROS production in *β*TC3 cells. Cells were pretreated with 10-40 *μ*M CHS or Gli for 24 h, followed by incubation with PA for 24 h, then intracellular ROS levels assayed with DHE and DCF-DA probe as described in the experimental section 4.5. Representative pictures from three independent experiments are shown (×400). MDA (b), SOD (c), GPx (d), and GSH (e) were measured after CHS and PA treatments by relative kits. The columns and errors bars are presented as means ± SEM (*n* ≥ 5). ^##^*P* < 0.01 compared with the CON group. ^∗∗^*P* < 0.01 compared with the PA group.

Intracellular ROS levels in *β*TC3 cells were measured by a fluorometric assay using DCFH-DA and DHE probes. As shown in Figure 5(a), intracellular ROS levels were increased dramatically in PA-treated *β*TC3 cells which showed as the fluorescent intensity was enhanced in the MOD group. When the *β*TC3 cells were treated with CHS, with the increased CHS concentrations, fluorescent intensity significantly decreased loaded with the DCFH-DA probe. The results demonstrated that the intercellular ROS in the cultured *β*TC3 cells could be downregulated by CHS. Similar results of CHS were found using the DHE fluorescence probe; the red fluorescence decreased with increasing concentration of CHS. Collectively, CHS treatment obviously suppressed intracellular ROS levels in PA-treated *β*TC3 cells.

To further detect whether CHS had effects on lipid peroxidation, we measured the MDA levels in *β*TC3 cells. The MDA level was significantly increased in the MOD group when treated with 0.5 mM PA; however, CHS at 10, 20, and 40 *μ*M significantly reduced the MDA concentrations (Figure 5(b)). Several enzymes were involved in the removal of ROS; among these, SOD constitute the first line of defense against oxidant stress. Based on the ROS-scavenging abilities of CHS, we assumed that the effect might be related to the expression of antioxidative enzymes. To investigate the mechanism underlying the protective effect of CHS, changes in the activities of SOD and GPx were measured in *β*TC3 cells. As shown in Figures 5(c)–5(e), SOD, GPx, and GSH activities were decreased in the MOD group when compared with the CON groups, whereas pretreatment with CHS significantly elevated SOD, GPx, and GSH activities. These results suggested that CHS could reverse PA-induced changes in the activities of antioxidant enzymes.

### 5.6. Effects of CHS on the Expression of Nrf2

**Figure 6:**
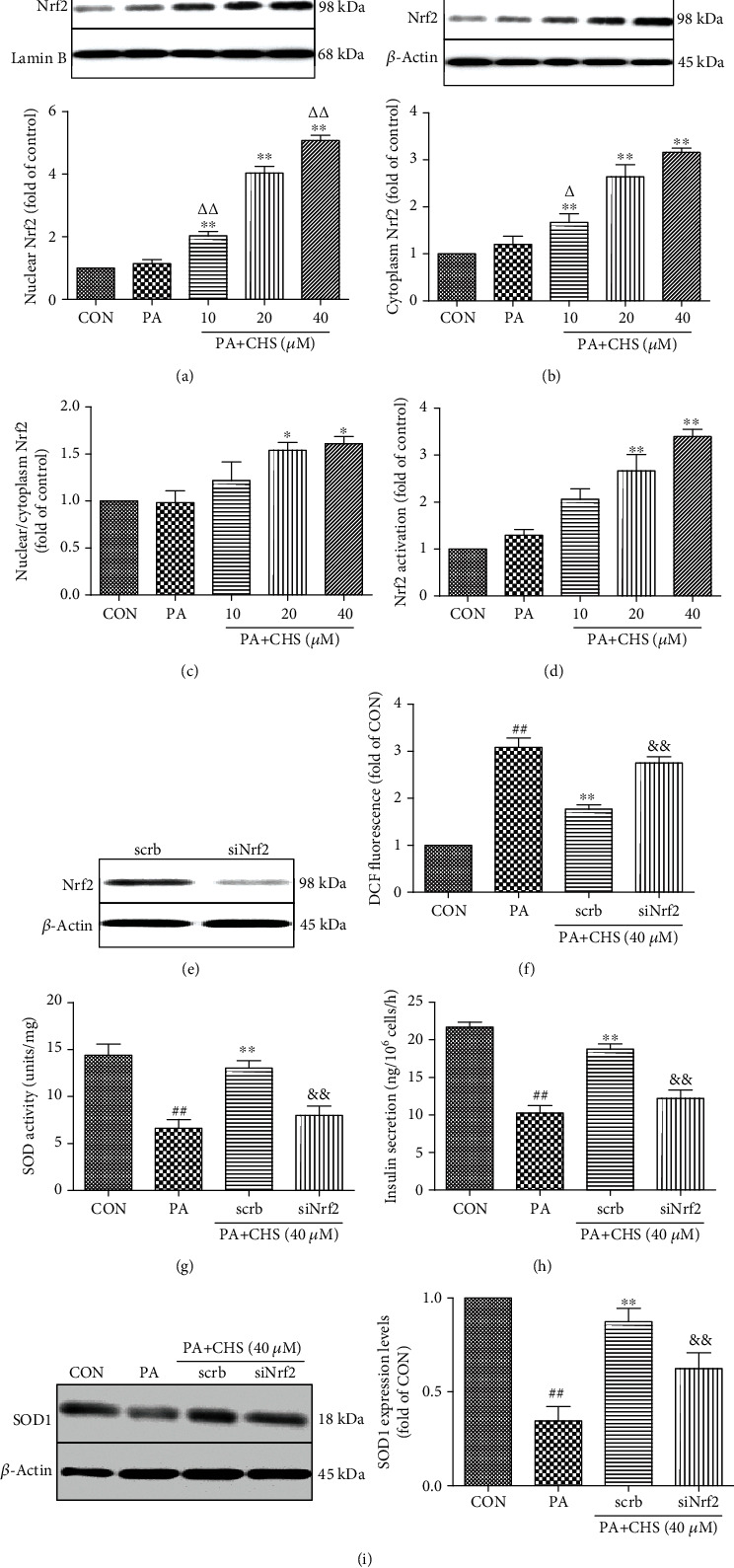
Effect of CHS on the expression of Nrf2 against lipotoxicity in *β*TC3 cells. *β*TC3 cells were incubated with 10-40 *μ*M CHS for 24 h then subjected to PA. The protein expression levels of Nrf2 in the nuclear protein (a) and cytoplasm (b) were measured by Western blotting. (a) Effects of CHS on the expression levels of Nrf2 in the nuclear protein; Lamin B was used as the internal control protein in the nuclear protein. (b) Effects of CHS on the expression levels of Nrf2 in the cytoplasm; *β*-actin was used as the internal control protein in the cytoplasm. (c) The ratio of nuclear/cytoplasm Nrf2 levels. (d) The Nrf2 transcriptional activity. (e) The *β*TC3 cells were treated with Nrf2-specific siRNA (40 nM) or scrb siRNA for 48 h, and Nrf2 expression levels in the cytoplasm were measured by Western blotting. Effects of siNrf2 on the levels of ROS (f), SOD (g), and insulin secretion (h), and the protein expression levels of SOD1 (i) were measured after different treatments. The columns and errors bars are presented as means ± SEM (*n* ≥ 5). ^##^*P* < 0.01 compared with the CON group, ^∗∗^*P* < 0.01 compared with the PA group, and ^&&^*P* < 0.01 compared with the scrb group. ^△△^*P* < 0.01 and ^△^*P* < 0.05 compared with the CHS (20 *μ*M) group.

To test the mechanism of the CHS, we assessed the activation of the Nrf2 pathway in *β*TC3 cells; the localization of Nrf2 was examined using an anti-Nrf2 monoclonal antibody. The Nrf2 levels in the cytoplasm and nuclear protein following treatment with 10, 20, and 40 *μ*M CHS for 24 h that had undergone lipotoxicity were detected by Western blotting. As shown in Figures 6(a) and 6(b), Nrf2 expression was slightly increased in response to PA, but compared with the PA group, CHS treatment significantly increased the expression and translocation of Nrf2 in *β*TC3 cells (Figures 6(a) and 6(b)). In addition, CHS increased the ratio of nuclear/cytoplasm levels (Figure 6(c)) and the Nrf2 transcriptional activity in the nuclear protein (Figure 6(d)).

Further studies showed that silencing of the Nrf2 transcription factor by using siRNA abolished the protective effects of CHS. As shown in Figures 6(e)–6(i), Nrf2 silencing blocked the effects of CHS on ROS and SOD levels; the protein levels of SOD1 and glucose-induced insulin secretion were also significantly reduced in *β*TC3 cells to the control group.

### 5.7. CHS-Activated IRS-2/Akt/GSK-3*β* Pathway in *β*TC3 Cells Exposed to Palmitate

**Figure 7:**
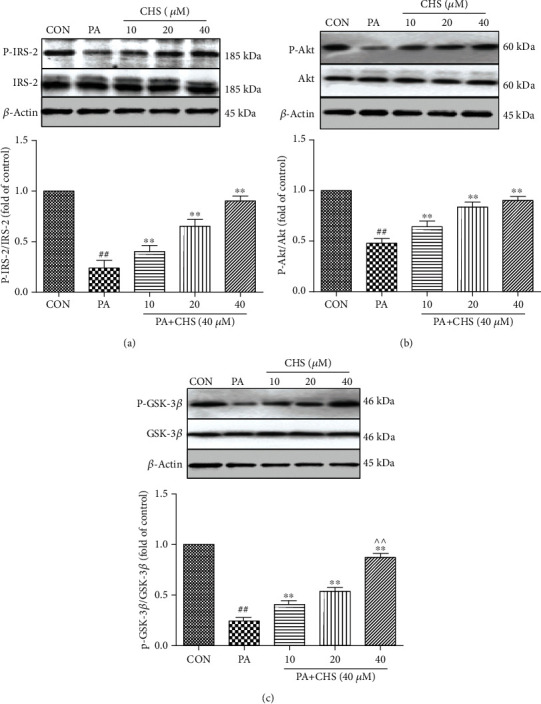
CHS induced the activation of IRS-2/Akt/GSK-3*β* signaling in PA-treated *β*TC3 cells. After being treated with CHS (24 h) and/or PA (another 24 h), the phosphorylation levels of IRS-2 (a), Akt (b), and GSK-3*β* (c) were examined. The columns and errors bars are presented as means ± SEM (*n* ≥ 5). ^##^*P* < 0.01 compared with the CON group. ^∗∗^*P* < 0.01 compared with the PA group. ^△△^*P* < 0.01 and ^△^*P* < 0.05 compared with the CHS (20 *μ*M) group.

The IRS-2 signaling pathway plays an important role in protecting *β*-cell viability and apoptosis. To further identify the effect of CHS against PA-induced apoptosis and dysfunction on the signal pathway, we next measured the activation of IRS-2/Akt/GSK-3*β* signaling using Western blotting analysis in *β*TC3 cells. Incubation of *β*TC3 cells in the presence of PA for 24 h led to a reduction in P-IRS-2; CHS incubation prevented PA-induced decrease in P-IRS-2 (Figure 7(a)). To investigate whether the restoration of P-IRS-2 by CHS subsequently improves its downstream signals, we assessed the activity of downstream proteins of IRS-2. Akt and GSK-3*β* in *β*TC3 cells were measured. As shown in Figures 7(b) and 7(c), CHS incubation resulted in enhancement of the phosphorylation of Akt at Ser473 and phosphorylation of GSK-3*β* at Ser9. These results suggested that CHS might protect PA-induced cell injury through the IRS-2/Akt/GSK-3*β* pathway.

### 5.8. CHS Prevented PA-Induced Apoptosis and Dysfunction via PI3K/Akt Pathway

**Figure 8:**
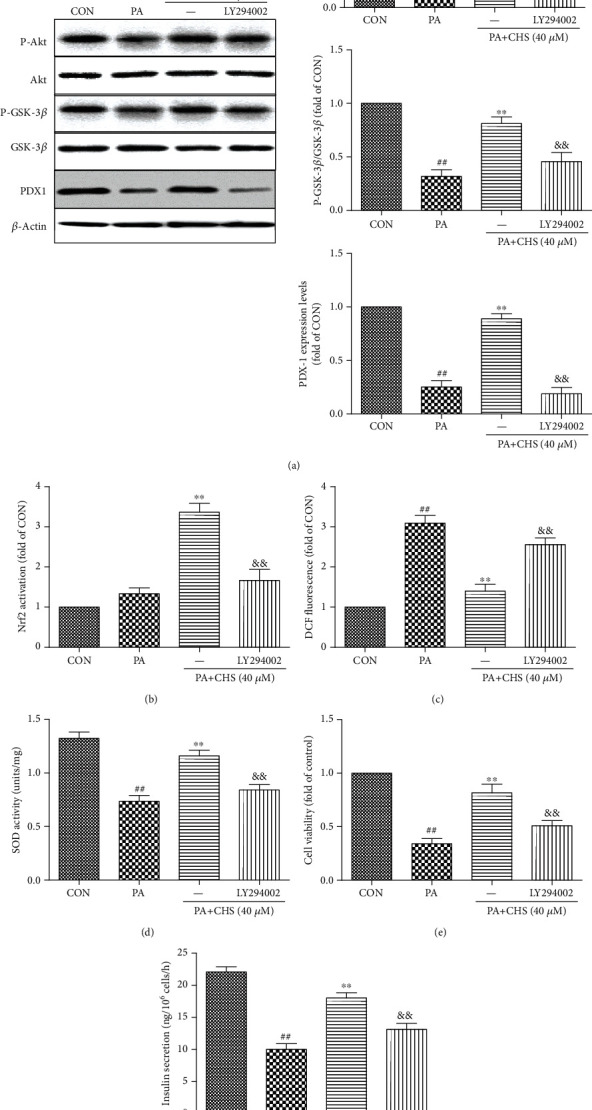
CHS protected against apoptosis of pancreatic *β*TC3 cells from lipotoxicity through the PI3K/Akt pathway. (a) *β*TC3 cells were pretreated with CHS (40 *μ*M) combined with or without LY294002 for 24 h, then incubated with PA (0.5 mM) for another 24 h. The cells were harvested, and protein levels were determined by Western blotting. Effects of LY294002 on Nrf2 transcriptional activity (b), ROS (c), SOD (d), cell viability (e), and insulin secretion (f) were measured after different treatments. The columns and errors bars are presented as means ± SEM (*n* ≥ 5). ^##^*P* < 0.01 compared with the CON group, ^∗∗^*P* < 0.01 compared with the PA group, and ^&&^*P* < 0.01 compared with the CHS treatment group.

To further investigate whether the activation of the PI3K/Akt signaling pathway is involved in the protective effect of CHS against PA-induced dysfunction in *β*TC3 cells, LY294002 was further used. When the PI3K/Akt pathway was inhibited by LY294002, CHS effects on Akt and GSK-3*β* phosphorylation (Figure 8(a)) and the Nrf2 transcriptional activity (Figure 8(b)) were blocked; its antioxidant activity was also significantly abolished by LY294002 treatment (Figures 8(c) and 8(d)). Further studies also found that CHS protection of *β*-cell survival and function, shown by increased cell viability and insulin secretion, was abolished when the PI3K inhibitor was present (Figures 8(e) and 8(f)).

### 5.9. Role of GSK-3*β* in PA-Induced *β*-Cell Apoptosis

**Figure 9:**
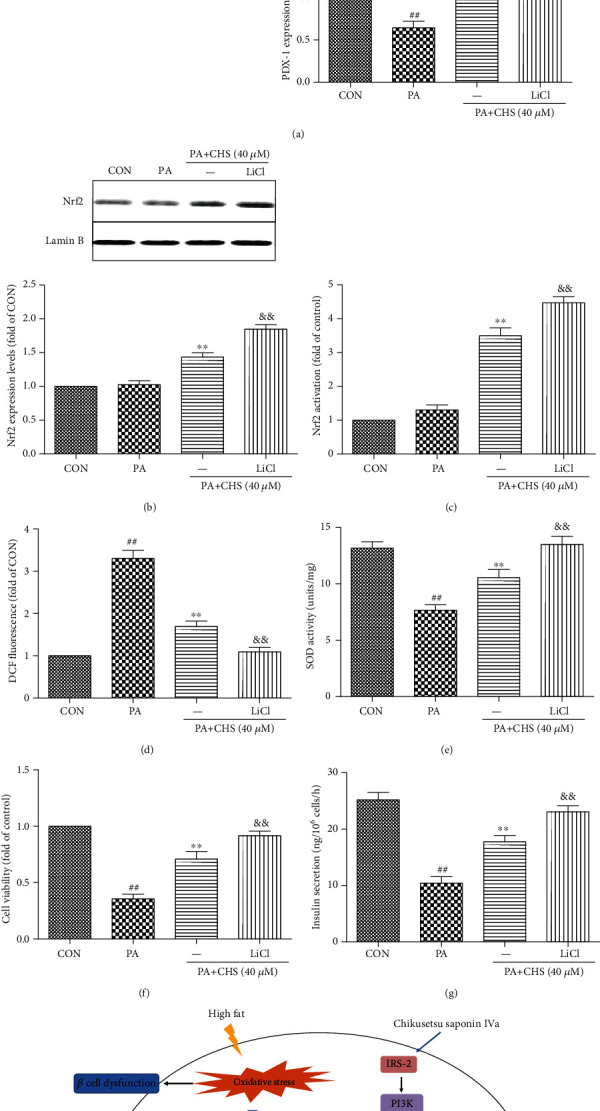
CHS protected against oxidative stress of pancreatic *β*TC3 cells from lipotoxicity through GSK-3*β* and the relationships between Nrf2 and GSK-3*β*. *β*TC3 cells were treated with CHS in the presence or absence of LiCl (GSK-3*β* inhibitor) for 24 h and then subjected to PA for another 24 h. Effects of LiCl (GSK-3*β* inhibitor) on CHS-induced GSK-3*β* phosphorylation and PDX-1 expression levels were measured (a). Immunoblot analysis showed the protein expression of Nrf2 in *β*TC3 cells pretreated with CHS (24 h) with or without PA (24 h) (b). Effects of LiCl on Nrf2 transcriptional activity (c), ROS (d), SOD (e), cell viability (f), and insulin levels (g) were measured after different treatments. The columns and errors bars are presented as means ± SEM (*n* ≥ 5). ^##^*P* < 0.01 compared with the CON group, ^∗∗^*P* < 0.01 compared with the PA group, and ^&&^*P* < 0.01 compared with the CHS treatment group. (h) Potential mechanism underlying the protective effects of CHS on lipotoxicity-induced cell injuries.

To further confirm the role of GSK-3*β* in mediating the CHS effects on *β*TC3 cells treated with PA in the presence or absence of the GSK-3*β* inhibitor (LiCl), the phosphorylation status of GSK-3*β* was measured. Western blotting results showed that CHS increased the phosphorylation of GSK-3*β* when PA is present, and LiCl further increased the expression levels of p-GSK-3*β* and PDX-1 (Figure 9(a)). The nuclear Nrf2 expression levels were also increased by LiCl+CHS treatment which was compared with the CHS treatment group (Figure 9(b)). As shown in Figures 9(d)–9(f), we found that cell viability, insulin secretion, and antioxidant activity were significantly increased by CHS compared with the PA group, which were dramatically enhanced in the presence of LiCl. Consistent with the above results, CHS increased the PDX-1 expression and nuclear translocation of Nrf2; the effects were increased when treated with LiCl (Figures 9(a) and 9(b)).

## 6. Discussion

Individuals with T2DM have higher levels of FFAs in circulation, and chronic exposure of *β*-cells to elevated levels of FFAs especially the saturated FFAs such as PA has been shown to induce *β*-cell apoptosis and dysfunction, thereby contributing to the pathogenesis of T2DM, which is defined as lipotoxicity [6, 27]. Oxidative stress has been implicated as an important mediator in the effector phase in lipotoxicity-induced *β*-cell apoptosis [28]. Reactive oxygen species (ROS), such as hydrogen peroxide (H_2_O_2_) and superoxide, are overproduced by palmitate subjection; meanwhile, antioxidative defense mechanisms in pancreatic *β*-cells are particularly weak and thus very vulnerable to ROS [29]. It is reported that increased accumulation of intracellular ROS led to *β*-cell dysfunction and apoptosis [30]. Thus, inhibiting oxidative stress may be a beneficial strategy for the treatment of T2DM.

CHS, one triterpenoid saponin with many pharmacological activities, is abundantly found in various medicinal plants, such as *Panax japonicus*, *Aralia taibaiensis*, and other plants. In previous studies, we had shown its protective effects against oxidative damage in cardiomyocytes and brain cells, and beneficial effects on intermittent hyperglycemia induced pancreatic islet injuries [24, 31, 32]. However, its effects against lipotoxicity-induced pancreatic islet injuries were largely unknown to us. In this study, the HFD-induced T2DM model and the PA-induced *β*-cell injury model were used to evaluate the effects of CHS.

It is well documented that long-term exposure to high levels of PA causes *β*-cell dysfunction characterized by reduced cell viability and insulin biosynthesis [33]. The dose of PA was based on the previous study which showed that 0.5 mM PA significantly reduced the beta cell viability (about 50% of normal cells) [34], different duration time and doses were measured in our previous study, and the results showed that the *β*TC3 cells treated with 0.5 mM PA for 24 h reached satisfactory results. CHS is a kind of oleanane-type saponin and has many similar features with oleanolic acid. Based on the toxicological study of oleanolic acid reported before and our toxicology results of CHS, 180 mg/kg had no obvious hepatorenal toxicity. Thus, CHS at a dosage of 45, 90, and 180 mg/kg were used in the animal study. The dosages used in the cell were based on the serum concentration of CHS in this study.

As the results showed, in diabetic rats, a long time HFD combined with STZ caused damage to the pancreatic tissue and reduced insulin secretion during OGTT, while CHS therapy markedly relieved it. In *β*TC3 cells, we substantiated that PA induced a significant reduction of cell viability and increased GSIS impairment and apoptosis; however, CHS reversed these injuries and restored the secretion activity under high FFA condition. Together, our study uncovered the prominent protective pancreatic effects of CHS against lipotoxicity *in vivo* and *in vitro*.

Oxidative stress has been well clarified in the pathogenesis of FFA-induced *β*-cell dysfunction [35]. However, the protective effect of CHS against PA-induced *β*-cell injury through regulating the antioxidant effect was largely unclear. In this study, *β*TC3 cells were stained with DHE and DCF-DA to measure the level of ROS in cells. Confocal imaging showed that 0.5 mM PA increased DHE and DCF fluorescence intensity and CHS treatment reversed these effects. Furthermore, antioxidant proteins affected by CHS were detected *in vivo* and *in vitro*. The results showed that CHS increased the levels of antioxidant proteins (SOD and GSH-Px) and decreased the levels of MDA in rat pancreatic tissues and *β*TC3 cells. These results indicated that CHS reduced the level of oxidative stress induced by FFA. Next, the possible mechanisms were studied in the further study.

Nrf2 is vital in antioxidative stress. Under basal conditions, Nrf2 is bound to Keap1 in the cytoplasm. When the cells are exposed to oxidative stress or other potentially damaging stimuli [36], Nrf2 released from Keap1 and transferred from the cytoplasm to the nucleus, which binds to the antioxidant response element (ARE), appears to be essential for the induction of phase 2 enzymes including SOD and GPx, thus reducing ROS and MDA content [37]. Activation of Nrf2 and its target genes may protect pancreatic *β*-cells from lipotoxicity [38]. According to the results of CHS on antioxidant proteins, we hypothesized that CHS had some effects on the Nrf2 pathway. As the results showed in this study, compared with the PA group, pretreatment of CHS increased the level of cytoplasm and nuclear Nrf2, together with its downstream SOD and GPx, indicating that CHS upregulated the Nrf2 expression and promoted its translocation into the nucleus. Furthermore, to illustrate whether the antioxidant effects of CHS were through Nrf2 nuclear translocation, a siRNA-targeted Nrf2 transfection experiment was performed. As shown in Figure 6, in siNrf2-transfected *β*TC3 cells, the Nrf2 protein in the nuclear protein was downregulated and the antioxidative and cytoprotective effects of CHS were both suppressed, indicating that the translocation of Nrf2 played an important role in the antioxidant capacity of CHS.

The insulin receptor substrate-2 (IRS-2) has important effects in regulating nutrient homeostasis and also shows an essential role in pancreatic *β*-cell function. In the liver, the deletion of IRS-2 induces glucose intolerance, and in the pancreas, the deletion of IRS-2 induces diabetes [39]. A previous study also showed that IRS-2 had protective effects against oxidative stress and mitochondrial dysfunction [40].

PI3K/Akt is considered as a critical downstream target of IRS-2 signaling in regulation of the pancreatic *β*-cell survival and function [41, 42]. PI3K/Akt has multiple functions, including glucose and lipid homeostasis, protein synthesis, and cell proliferation [43]. The damage of PI3K/Akt leads to insulin resistance which induces T2DM further, and in turn, insulin resistance causes further impairment to PI3K/Akt, which is a vicious circle [44]. Thus, IRS-2/PI3K/Akt has been a target for the development of drugs used to treat obesity or T2DM. To explore the protective effects of CHS on *β*-cells, the phosphorylation levels of IRS-2 and Akt were measured. The results showed that PA-induced lipotoxicity dramatically inhibited IRS-2 and Akt phosphorylation, while coincubation with CHS reversed these downregulations under lipotoxicity condition. Inhibition of Akt by LY294002 blocked the protective effects of CHS in *β*TC3 cells. Accordingly, the data suggested that the protection of CHS from PA-induced lipotoxicity might be initiated via activation of the IRS-2/PI3K/Akt pathway.

GSK-3*β* is a well-defined downstream target of the IRS-2/PI3K/Akt signaling pathway; its activity is inactivated by phosphorylation of serine. IRS-2 deficiency causes a reduction in the inhibition of GSK-3*β* by its phosphorylation; upregulation of GSK-3*β* activity reduced islet *β*-cell mass and the resultant diabetic phenotype emerged in mouse models with deficiency in receptors for insulin or IRS-2 [45]. Previous studies also showed that inhibition of GSK-3*β* has been associated with the survival mechanism against various stresses including oxidative stress [46] and enhances the expression of proapoptotic proteins [47]. In this study, we found that PA increased the activity of GSK-3*β* by inhibiting its phosphorylation and was inhibited in the presence of CHS. Furthermore, we found that the effects of CHS were the same as the GSK-3*β* inhibitor (LiCl), and they restored the PA-induced reduction of PDX-1, cell viability, and insulin release together. In Akt inhibitory experiments, we found that the phosphorylation effect of GSK-3*β* by CHS was inhibited by LY294002 treatment, indicating that GSK-3*β* inactivation by CHS was dependent on the Akt activation.

The presented results addressed the question in which the upstream mediated by CHS contributes to its effects on Nrf2 regulating antioxidant defenses. In both GSK-3*β* inhibitory and Akt inhibitory experiments, we found that the ROS and SOD levels were affected by LiCl and LY294002. From the literatures, we found that GSK-3*β* is a negative regulator of Nrf2. GSK-3*β* induces Nrf2 phosphorylation, nuclear exclusion, and degradation of Nrf2, which inactivates the Nrf2 transcriptional activity. Studies have also shown that inhibition of GSK-3*β* exerts cell protection though the direct regulation effects on the Nrf2 pathway [46]. Thus, we assumed that the effect of CHS on Nrf2 was through regulating the GSK-3*β*. As expected, we found that blockade of GSK-3*β* activation by LiCl increased the induction effects of CHS on Nrf2 nuclear translation, and the protective effects of CHS were also strengthened by LiCl. These results suggested that GSK-3*β* was involved in the regulation effects of CHS on the Nrf2-relating antioxidant system.

In conclusion, we demonstrated for the first time that CHS protected pancreatic *β*TC3 cells from PA-induced apoptosis and dysfunction through IRS-2/Akt/GSK-3*β*, associated with the activating Nrf2 pathway and attenuation of ROS generation (Figure 9(g)).

## Data Availability

The data used to support the findings of this study are available from the corresponding author upon request.
